# Adjusting droxidopa for neurogenic orthostatic hypotension in a patient with Parkinson disease

**DOI:** 10.1007/s10286-016-0389-z

**Published:** 2017-06-19

**Authors:** Brent P. Goodman, Daniel Claassen, Ali Mehdirad

**Affiliations:** 1 Department of Neurology, Mayo Clinic Arizona, Scottsdale, AZ USA; 2 Department of Neurology, Vanderbilt University, Nashville, TN USA; 3 Cardiac Electrophysiology Section, Division of Cardiology, Department of Internal Medicine, Saint Louis University, St. Louis, MO USA

**Keywords:** Neurogenic orthostatic hypotension, Droxidopa, Midodrine, Parkinson’s disease

## Challenge question

How does supine hypertension influence the management of neurogenic orthostatic hypotension?

## Case presentation

Mrs. T is a 68-year-old woman with Parkinson disease (PD). She lives at home with her husband of 40 years. She is ambulatory without assistance and remains physically active, going for short walks every day. Her past medical history is significant for hypertension, hyperlipidemia, and osteoporosis. Her current medications include Sinemet^®^ (carbidopa/levodopa 25 mg/100 mg) taken orally (po) three times daily (TID), ropinirole XL 2 mg po once daily (QD), pravastatin 10 mg po QD, metoprolol XL 25 mg po QD, and chlorthalidone 25 mg po QD.

Approximately 6 months ago, she presented with recurrent symptoms of dizziness and feeling as if she might faint. She had at least three episodes before seeking care approximately 2 months ago. At that time, she was diagnosed with symptomatic neurogenic orthostatic hypotension (nOH), her chlorthalidone treatment was discontinued, and she was started on midodrine 5 mg po TID. Her nOH symptoms resolved.

She visits the office for follow-up after beginning midodrine treatment for nOH. She and her husband report that she continues to do well on midodrine and she has not had any further episodes of dizziness, fainting, or falls. However, she reports experiencing an “itchy scalp” that is bothersome. Her blood pressure (BP) is monitored and recorded at home. On review by her neurologist, it is noted that her initial BP in the morning prior to getting out of bed has been very elevated—typically around 190/110 mmHg—and that it decreases to 130/70 mmHg within 3 min of standing. Her examination is significant for masked face, mild bilateral hand tremors, stooped posture, and a shuffling gait.

## Expert commentary (Dr. Goodman)

Supine hypertension is a common feature of autonomic failure in PD and multiple system atrophy (MSA). In a recent study, supine hypertension was seen in 34% of 197 PD patients and 37% of 78 MSA patients, and the presence of supine hypertension was independent of age, disease duration, or stage of disease [1, 4]. In PD and MSA, supine hypertension often occurs in conjunction with OH, further complicating the management of these patients. Typically, supine hypertension is asymptomatic, and is therefore most commonly first recognized after patients become aware of a problem with BP control and start checking their BPs more frequently. Nonpharmacologic measures (such as the liberalization of salt and fluid intake) and pharmacotherapies used to treat OH may exacerbate—but do not necessarily cause—supine hypertension.

## Expert commentary (Dr. Claassen)

Supine hypertension is a paradoxic phenomenon where the underlying mechanisms are poorly understood. Supine hypertension limits treatment options for nOH and has been associated with several systemic complications. Many times, the concern when treating supine hypertension relates to cerebral bleeds, but the main clinical problems from supine hypertension include impaired renal function [2] and left ventricular hypertrophy [5]. Clinically, patients are most concerned about nocturnal diuresis, in which they can diurese liters overnight. If nonpharmacologic measures are not sufficient to relieve supine hypertension, I recommend administration of a short-acting BP-lowering agent at bedtime. Such agents typically include transdermal nitroglycerin, captopril, hydralazine, clonidine, and losartan, which, when used at bedtime, can prevent nocturnal supine hypertension [3].

## Case continuation

It is rewarding clinically to recognize and effectively treat supine hypertension, and to educate patients. The easiest way to treat supine hypertension is to avoid lying down fully supine. For Mrs. T, it is good to remind her that, when taking midodrine, she should not lie down for at least 4 h. Eating before bedtime results in increased blood flow to the digestive tract and is a good nonpharmacologic treatment for supine hypertension. Also, elevating the head of the bed by approximately 30° can help to lower BP at night and decrease nocturnal diuresis. With these in mind, the patient is instructed to elevate the head of the bed 6–9 inches, avoid drinking water within an hour of bedtime, eat a snack prior to bedtime, and avoid compression garments while supine and in bed. In addition, the patient is instructed to avoid nasal decongestants, nonsteroidal anti-inflammatory drugs, and other over-the-counter medications that may have the potential to raise BP near bedtime.

Pharmacologic agents to treat symptomatic nOH, such as midodrine and droxidopa, should be utilized during daytime hours when symptoms of OH are more likely to be most severe; avoid or minimize doses in the afternoon or evening to reduce the degree of supine hypertension exacerbation that may occur with these medications, especially at bedtime [3]. Therefore, for this patient, her midodrine dosage was reduced to a modified twice-daily dosing: in the morning and at midday.

In this case scenario, the patient had reduced nOH symptoms with midodrine treatment but had significant supine hypertension as well as a bothersome itchy scalp. After educating the patient regarding the nonpharmacologic methods of reducing supine hypertension, the patient returned for another follow-up visit. The nonpharmacologic interventions and medication adjustments did not substantially impact the degree of supine hypertension in this patient, and she continues to report a bothersome itchy scalp.

At this point, stopping midodrine and switching to droxidopa could be considered. The mechanisms involved in supine hypertension are not precisely known, and likely differ in the various forms of autonomic failure. Early experience suggests that, just as midodrine and droxidopa are not identical in how they impact OH, they may also influence supine hypertension differently. However, the main reason for switching from midodrine to droxidopa in this patient is related to the reduction in the adverse events of piloerection and itchy scalp.

## Expert commentary (Dr. Mehdirad)

Piloerection and itchy scalp are well-described and rather common adverse events associated with midodrine. If an itchy scalp were bothering the patient, I would switch to droxidopa. Midodrine is not FDA approved for nOH, while droxidopa is the only FDA-approved medication for nOH. Additionally, piloerection and itchy scalp are not adverse events associated with droxidopa. As such, I would treat nOH in this patient preferably with droxidopa only.

## Case continuation

If midodrine is causing bothersome side effects, then it is recommended to try treatment with droxidopa. After switching, the patient and caregiver should be instructed to continue careful monitoring of orthostatic BP readings and, importantly, to determine if the patient notes clinical improvement under the new regimen.

In this case scenario, the patient is prescribed droxidopa starting at 100 mg on a modified TID dosing schedule in which the patient takes the medication at approximately 8am, noon, and 4 pm; she is instructed to uptitrate the dose by 100 mg on this modified TID schedule every 24–48 h until remission of her nOH symptoms or up to a maximum dosage of 600 mg TID. This patient requires 600 mg on the modified TID schedule to achieve symptomatic improvement. Since this treatment, she has reported much fewer episodes of feeling faint or dizzy for 2 months and the itchy scalp resolved completely after discontinuation of midodrine. Her supine BP is now 170/85 mmHg (prior to arising from bed in the morning) and 130/70 mmHg after 3 min upon standing, and she is asymptomatic.
